# Outcomes of Kidney Transplantation from Deceased Donors with Severe Acute Kidney Injury (AKIN Stage 3): A Preliminary Single-Centre Analysis

**DOI:** 10.3390/medsci13030188

**Published:** 2025-09-14

**Authors:** Juan A. Encarnación, Elisabeth Coll, Clara Manso, Santiago Llorente, Francisco Morales, Isabel Saura, Pedro López-Cubillana, Pablo Luis Guzman Martínez-Valls, Gloria Martínez, Isabel De la Fuente, Enrique Cárdenas, Jose L. Alonso-Romero, Paula Ruiz, José Moya, Beatriz Domínguez-Gil, Mario Royo-Villanova

**Affiliations:** 1 Department of Radiation Oncology, Virgen de la Arrixaca University Clinical Hospital, 30120 Murcia, Spain; isabeldelafuente123@gmail.com (I.D.l.F.); ecarcan@gmail.com (E.C.); 2 Murcian Institute of Biosanitary Research, 30120 Murcia, Spain; clara92mm@hotmail.com (C.M.); josel.alonso2@carm.es (J.L.A.-R.); dm.oncoarrixaca2@gmail.com (P.R.); mariorvr@hotmail.com (M.R.-V.); 3 Transplant Coordination, Virgen de la Arrixaca University Clinical Hospital, 30120 Murcia, Spain; jose.moya0185@gmail.com; 4 National Transplant Organization (ONT), 28049 Madrid, Spain; ecoll@sanidad.gob.es (E.C.); bdominguez@sanidad.gob.es (B.D.-G.); 5 Department of Intensive Care Medicine, Virgen de la Arrixaca University Clinical Hospital, 30120 Murcia, Spain; 6 Department of Nephrology, Virgen de la Arrixaca University Clinical Hospital, 30120 Murcia, Spain; sllorentev@telefonica.net (S.L.); fmorales542@gmail.com (F.M.); isaslujan@hotmail.com (I.S.); 7 Department of Urology, Virgen de la Arrixaca University Clinical Hospital, 30120 Murcia, Spain; pedrolopezcubillana@gmail.com (P.L.-C.); pabloguzmanmv@gmail.com (P.L.G.M.-V.); gloriamartinez2010@gmail.com (G.M.); 8 Department of Medical Oncology, Virgen de la Arrixaca University Clinical Hospital, 30120 Murcia, Spain

**Keywords:** kidney transplantation, acute kidney injury, AKIN 3, delayed graft function, donor selection, graft survival

## Abstract

Background: The shortage of donor kidneys has prompted interest in using organs from donors with severe acute kidney injury (AKI), but robust data on outcomes from donors with AKIN stage 3 remain limited. Methods: This single-centre, retrospective cohort study compared outcomes of kidney transplants from deceased donors with AKIN stage 3 AKI to matched non-AKI donors (*n* = 57 per group; matched by donor age ±5 years, year of transplant, and major cardiovascular risk factors). Primary outcomes were delayed graft function (DGF), death-censored graft survival, and patient survival. Secondary outcomes included renal function at follow-up. Results: DGF occurred in 54.4% (31/57) of AKIN 3 recipients vs. 33.3% (19/57) of non-AKI recipients (risk difference 21.1%, 95% CI 3.1–39.2; *p* = 0.037). Five-year death-censored graft survival was 94.7% vs. 96.4% (HR 1.28, 95% CI 0.25–6.52; *p* = 0.645). Five-year patient survival was 84.8% vs. 84.0% (HR 0.96, 95% CI 0.30–3.05; *p* = 0.979). Median follow-up was 32 months. Conclusions: In this preliminary, selected kidneys from AKIN stage 3 donors yielded similar medium-term graft and patient survival to non-AKI donors, despite higher DGF incidence. Findings should be interpreted cautiously and confirmed in adequately powered, multicentre studies with extended follow-up.

## 1. Introduction

Kidney transplantation using organs from deceased donors with acute kidney injury (AKI) has been a subject of considerable interest within the scientific community, with numerous studies evaluating the risks and benefits of this approach [1,2,3,4,5,6,7,8,9,10,11,12]. Kidney transplantation remains the treatment of choice for patients with end-stage renal disease, offering improved quality of life and greater survival compared to dialysis [13,14]. However, the limited availability of donor organs continues to pose a significant challenge, prompting the medical community to explore various strategies to expand the donor pool. One such strategy is the utilization of kidneys from donors with acute kidney injury (AKI). Despite growing evidence suggesting that these organs may yield satisfactory outcomes, concerns persist regarding their long-term viability and the potential risk of graft dysfunction.

Multiple studies in the scientific literature have assessed the impact of AKI in deceased donors and its association with graft outcomes in recipients. These studies have reported variable results: some indicate that donor AKI does not significantly affect graft survival, whereas others suggest higher rates of delayed graft function or acute rejection. However, no study has definitively clarified these outcomes. Moreover, most of the available literature includes donors with varying degrees of AKI, making it difficult to accurately determine the impact of each AKI stage on transplant outcomes [6,7,8,9,10].

The aim of our study is to investigate kidney transplant outcomes by focusing exclusively on cases involving donors with the most severe degree of acute renal dysfunction—AKIN stage 3—according to the Acute Kidney Injury Network (AKIN) classification.

Our underlying hypothesis is that, despite the severity of renal injury at the time of donation, the acute and potentially reversible nature of the damage allows for functional recovery of the graft post-transplantation, leading to outcomes comparable to those of transplants from donors without AKI in terms of graft function and recipient survival.

Acute kidney injury was defined and staged according to the Acute Kidney Injury Network (AKIN) criteria [15]. AKI is defined as an increase in serum creatinine of ≥0.3 mg/dL or ≥50% within 48 h, or urine output < 0.5 mL/kg/h for more than 6 h.

- Stage 1 is defined as an increase in serum creatinine of ≥0.3 mg/dL or 150–200% from baseline, or urine output < 0.5 mL/kg/h for 6 to 12 h.
- Stage 2 is defined as an increase in serum creatinine of 200–300% from baseline, or urine output < 0.5 mL/kg/h for 12 to 24 h.
- Stage 3, which defines the AKI group in our study, is characterized by an increase in serum creatinine > 300% from baseline, or an absolute creatinine level ≥ 4.0 mg/dL with an acute rise of at least 0.5 mg/dL, or urine output < 0.3 mL/kg/h for more than 24 h, anuria for more than 12 h, or initiation of renal replacement therapy.

To evaluate this hypothesis, we designed a comparative study involving two well-defined cohorts: one comprising only donors with AKIN stage 3 and the other consisting of donors without AKI and with preserved renal function, matched by donor age and year of transplantation. Through this comparative analysis, we aim to provide evidence that can inform organ selection criteria and help reduce the discard rate of potentially transplantable kidneys.

This work is designed as an exploratory, hypothesis-generating study aimed at providing preliminary evidence to guide the design of adequately powered, multicentre investigations with longer follow-up.

### Rationale of the Study

Several studies have explored the feasibility of using kidneys from donors with AKI, assessing graft survival and clinical outcomes in recipients. A recent meta-analysis [5] reviewed the safety of kidney transplantation from AKI donors and concluded that, overall, these kidneys can be used without significantly compromising graft survival. Other studies have found that acute kidney injury in the donor is not directly associated with allograft failure [6,7,8], whereas some reports have identified a potential association between donor AKI and an increased risk of acute rejection in recipients [2].

Multicenter cohort studies have evaluated the association between donor AKI and graft function, yielding mixed results in terms of renal recovery and rejection incidence [3,4,9].

Some investigations have challenged the routine exclusion of kidneys from donors with severe AKI, highlighting the opportunity to expand the donor pool and reduce the discard rate of viable organs [7,8,11,12].

Despite several studies evaluating outcomes of kidney transplantation from donors with varying degrees of acute kidney injury, no controlled studies have specifically and exclusively addressed the outcomes of recipients from donors with AKIN stage 3 AKI. This knowledge gap is clinically relevant, given the potential to expand the donor pool while ensuring acceptable graft and patient outcomes. The present study aims to provide preliminary, hypothesis-generating data on this specific donor subgroup.

Our study aims to specifically analyze outcomes from donors with severe renal failure (AKIN 3) and compare them to a reference population of donors without AKI. By doing so, we aim to provide a more detailed understanding of the impact of severe acute kidney injury on graft function.

## 2. Materials and Methods

We conducted a retrospective analysis of a prospectively collected database of patients who underwent kidney transplantation at Hospital Clínico Virgen de la Arrixaca between 1 January 2016, and 1 April 2024. Data were obtained from electronic medical records with approval from the hospital’s Institutional Review Board. The inclusion criterion was all recipients of kidney transplants from deceased donors who met AKIN stage 3 criteria. For comparison purposes, a control group was selected consisting of transplant recipients from the same period, matched by age and cardiovascular risk factors (CVRF), who had received kidneys from donors without acute kidney injury.

Matching was performed in a 1:1 ratio based on donor age (±5 years), year of transplantation, and major cardiovascular risk factors (history of hypertension, diabetes, dyslipidaemia, and smoking status).

Other potential confounding variables, including donor comorbidities (e.g., chronic kidney disease, cardiovascular disease), cause of death and details of perioperative management (ischaemia times), were recorded where available but were not included in the matching process or adjusted for in the primary analysis. This limitation should be considered when interpreting the results. Exclusion criteria included recipients of kidney transplants from living donors, simultaneous kidney-pancreas transplants, and other multiorgan transplants.

The variables analyzed included clinical characteristics of donors and recipients, focusing on serum creatinine and estimated glomerular filtration rate (eGFR) measured with CKD-EPI, both at baseline and at the time of donation. We also assessed delayed graft function (DGF), one-year graft survival, and all-cause patient mortality. DGF was defined as the requirement for at least one dialysis session within the first seven days post-transplantation, in the absence of reversible or extrarenal causes (e.g., hyperkalemia, fluid overload, or surgical complications). None of the donors included in the cohort were receiving renal replacement therapy (dialysis) at the time of procurement. Cold ischemia time (CIT) and warm ischemia time (WIT) were recorded for all transplant procedures. Cold ischemia time was defined as the interval between cross-clamping in the donor and reperfusion in the recipient. Warm ischemia time was defined as the time between removal from cold storage and reperfusion of the graft. In all cases involving donors, a preimplantation kidney biopsy was performed prior to organ acceptance. The histological evaluation was conducted by experienced nephropathologists and followed established criteria to assess glomerular, tubular, interstitial, and vascular integrity. The median follow-up duration was 32 months, censored for death.

The sample size was determined by the availability of eligible cases within the study period rather than a priori power calculation; therefore, the statistical power to detect clinically relevant differences is limited, and the results should be interpreted with caution.

### 2.1. Statistical Analysis

Continuous variables were presented as means ± standard deviation or medians (interquartile range), as appropriate. Categorical variables were reported as absolute and relative numbers. The primary objectives were to compare baseline characteristics, DGF, and patient and graft survival rates between the study groups. Depending on the distribution of the variable, Student’s t-test, Mann–Whitney U test, or median test was used in univariate analysis for quantitative variables, and the chi-square test for categorical ones.

Patient and Graft (Death-Censored) Survival were Analyzed Using the Kaplan–Meier Method. Survival was not Calculated When *n* < 10.

### 2.2. Ethics

The study was conducted in accordance with the ethical principles outlined in the Declaration of Helsinki and was approved by the Ethics Committee of Arrixaca hospital (CEIm), with approval number [HCUVA-10-2024]. All participants provided written informed consent to participate in this study. For deceased donors, consent for organ donation was obtained from the donor’s next of kin in accordance with national regulations. Kidney recipients provided written informed consent to participate in this study.

## 3. Results

A total of 114 recipients from 66 donors, all performed at the same transplant centre were included in the study.

The characteristics of the donors and recipients in both groups are presented in Table 1 and Table 2. 59.4% met the criteria for AKI stage 3 based on serum creatinine.

Follow-up time analysis revealed a median follow-up duration of 32 months in both groups. The interquartile range (IQR) was 48 months, reflecting the spread of follow-up. The minimum follow-up was 1 month, and the maximum reached 103 months.

**Figure 1:**
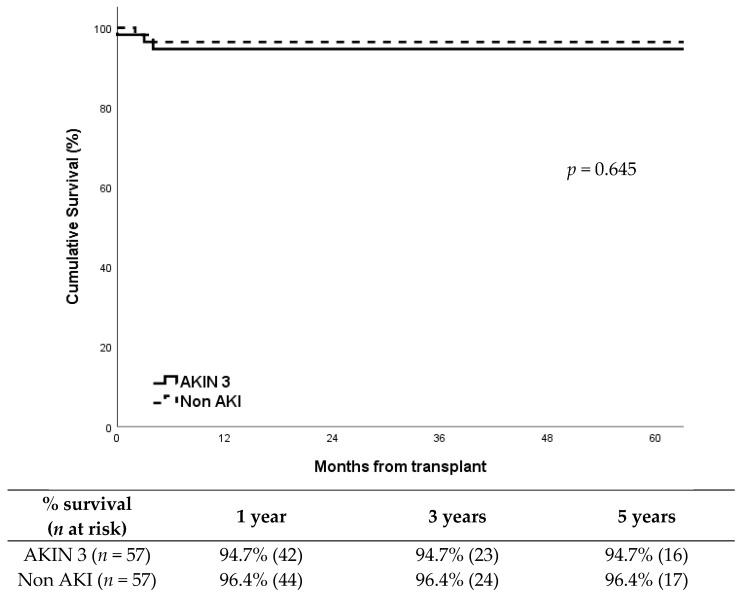
Death censored graft survival according to study group.

DGF occurred in 54% (31/57) of recipients in the AKIN 3 group compared to 33% (19/57) in the non-AKI group, showing a significantly higher incidence in the AKIN 3 group (*p* = 0.037). Despite this, death censored graft survival showed no differences between groups (*p* = 0.645), around 95% at five years after transplantation in both groups (Figure 1). Cold ischemia time was similar between groups: median CIT was 13.4 h (IQR: 3.1) in the AKIN 3 group vs. 13.1 h (IQR: 3.0) in the non-AKI group (*p* = 0.631). Warm ischemia time showed no significant differences either: median WIT was 32 min (IQR: 6) vs. 31 min (IQR: 5), respectively (*p* = 0.472).Graft losses were all in the first year; in the AKIN 3 group (*n* = 3) were due to refractory acute rejection, renal vein thrombosis, and chronic BK virus nephropathy; in the non-AKI group (*n* = 2), causes were refractory acute rejection and progressive chronic nephropathy.

**Figure 2:**
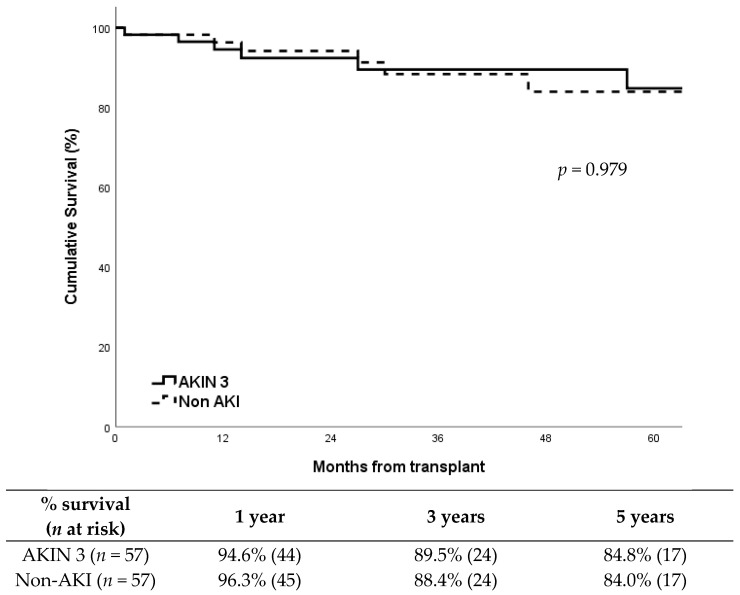
Patient survival according to study group.

Patient survival was also very similar between groups (Figure 2; *p* = 0.979), with a five-year survival around 85%. The causes of death including: AKIN 3 group: acute myocardial infarction (2), pneumonia (1), unexpected cardiorespiratory arrest (1), Klebsiella pneumoniae bacteremia (1), cerebral hemorrhage (1), and COVID-19 (1). Non-AKI group:acute myocardial infarction (2), pneumonia (1), sepsis secondary to fungal infection (1), traumatic brain injury (1), and pulmonary embolism (1).

## 4. Discussion

This study provides evidence supporting the safety and feasibility of using kidneys from deceased donors with acute kidney injury (AKI) classified as AKIN stage 3 and with eGFR < 60 mL/min/1.73 m^2^, by comparing their outcomes to those of kidneys from donors without AKI. Although donor AKI has traditionally been regarded as a risk factor for poorer post-transplant outcomes, our findings suggest that, with rigorous and appropriate donor selection, kidney grafts from AKIN 3 donors may represent a viable alternative without significantly compromising graft function or patient survival.

### 4.1. Comparison with Previous Literature

Our results are consistent with previous studies that have examined the association between donor AKI and post-transplant outcomes [3,4,5]. Prior research has demonstrated that kidneys from donors with AKI can recover function after transplantation and that short-and medium-term graft survival is comparable to that of grafts from non-AKI donors [15]. A recent meta-analysis concluded that donor AKI does not significantly affect graft survival [5]. Other studies have found that while AKI may increase the risk of delayed graft function, this does not necessarily translate into inferior long-term survival [6,7].

However, most of these studies include donors across various stages of AKI and do not focus specifically on those classified as AKIN stage 3, limiting the ability to accurately assess the impact of severe acute kidney injury [2,3,4]. Our study stands out by exclusively analyzing transplants from AKIN 3 donors and comparing them with a matched cohort of non-AKI donors, allowing for a more precise evaluation of the effect of severe acute renal failure on transplant outcomes.

### 4.2. Impact on Graft Function

One of the main findings of our study is that one-year graft function in recipients of kidneys from AKIN 3 donors was comparable to that observed in recipients of kidneys from non-AKI donors. The median serum creatinine at follow-up was 1.39 mg/dL in the AKIN 3 group versus 1.28 mg/dL in the non-AKI group, suggesting adequate renal function recovery in most patients. These findings support the hypothesis that, in the absence of pre-existing chronic kidney disease, kidneys from donors with severe AKI can recover function after transplantation, yielding satisfactory long-term outcomes.

However, the incidence of delayed graft function was significantly higher in the AKIN 3 group (55%) compared to the non-AKI group (34%), aligning with previous literature describing an increased risk of DGF in grafts from AKI donors [8]. Despite this, the majority of patients with DGF eventually recovered renal function, indicating that initial DGF should not be regarded as a predictor of long-term graft failure. This result underscores the importance of optimal perioperative management in patients experiencing DGF to support renal recovery.

### 4.3. Graft and Patient Survival

One-year graft survival in the AKIN 3 group was 94.7%, with only three graft losses, all occurring in donors over 65 years of age. In comparison, one-year graft survival in the non-AKI group was 96.4%, with two graft losses. These findings indicate that donor AKI is not associated with reduced graft survival, reinforcing the viability of using kidneys from donors with severe AKI. Previous studies [16] have reported similar graft loss rates in standard criteria donor transplants, suggesting that organ acceptance criteria could be made more flexible without compromising clinical outcomes.

Regarding overall mortality, rates were 12.5% in the AKIN 3 group and 10.7% in the non-AKI group. The causes of death were not directly related to graft function. Most deaths were attributed to cardiovascular events, infections, and other causes unrelated to the transplant itself, suggesting that mortality in these patients is more closely linked to pre-existing comorbidities than to graft quality. These survival data for donors with AKIN 3 are comparable to the 5-year survival rates reported for donors, in general, in published global survival studies [17].

### 4.4. Advanced Donor Age as a Risk Factor for Graft Loss

One of the observations in our cohort was that all three cases of graft loss occurred in recipients of kidneys from donors over 65 years of age. While this may suggest a potential association between advanced donor age and inferior outcomes, this finding must be interpreted with caution given the very limited number of events. The use of a fixed age cutoff (>65 years) oversimplifies a multifactorial issue. Advanced donor age should not be viewed as an isolated risk factor, but rather as a potential surrogate marker for underlying chronic kidney damage, which may be exacerbated when superimposed by severe acute kidney injury (AKI), a phenomenon akin to acute-on-chronic injury. In this context, the resilience of aged kidneys to recover from AKI is likely diminished. Distinguishing between purely acute injury and acute-on-chronic damage is essential, and this differentiation requires preimplantation histological assessment, which was not systematically performed or reported in our study. Therefore, categorical conclusions against the use of older donors are unwarranted. Instead, we propose that donor age be considered within a broader clinical and histopathological evaluation framework, highlighting the need for routine biopsy in marginal grafts to guide clinical decision-making more accurately [18,19,20]. Taken together, these findings highlight the complex interplay between donor age, pre-existing chronic injury, and superimposed acute kidney injury. However, the lack of immunological data, procurement details, and formal age-stratified analyses restricts the ability to draw definitive conclusions. Future research integrating these variables will be crucial to refine risk stratification and optimize donor selection criteria.

### 4.5. Clinical Implications and Future Research Directions

This study has important clinical implications for kidney transplant organ selection. Our findings support the inclusion of kidneys from AKIN stage 3 donors in transplant programmes, which could help reduce organ discard rates and expand the available donor pool. However, rigorous donor evaluation remains essential to ensure that acute renal dysfunction is not secondary to an underlying chronic kidney disease that could compromise graft recovery post-transplantation.

Future research should include multicenter studies with larger patient cohorts to validate these findings and assess long-term outcomes. Additionally, the incorporation of renal injury biomarkers may help better predict the recovery potential of these grafts, ultimately improving organ selection criteria and allocation strategies.

### 4.6. Clinical Relevance

Our findings suggest that, in this cohort, the use of kidneys from donors with stage 3 AKI was associated with similar medium-term graft function and patient survival compared to non-AKI donors, although the observational design and sample size preclude definitive conclusions. Post-transplant outcomes suggest that, under appropriate conditions, these organs can be successfully integrated into recipients, supporting their consideration in clinical practice.

In comparison with previous literature, this study reinforces and complements prior research and meta-analyses, offering a focused and detailed analysis of donors with AKIN 3 [1,2,3,4,5]. The uniqueness of this work lies in its exclusive focus on this specific donor subgroup, enabling a deeper understanding of its clinical impact and safety in kidney transplantation.

Furthermore, the study offers a detailed evaluation of graft function, including quantitative parameters such as post-transplant serum creatinine levels and the incidence of delayed graft function. These data provide key information for clinical decision-making in both organ selection and the postoperative management of transplant recipients.

The assessment of graft and patient survival revealed comparable outcomes between the two groups studied, reinforcing the robustness of our results and their applicability to clinical settings. The evidence suggests that, in terms of function and medium-term prognosis, grafts from AKIN 3 donors can be safely incorporated into organ allocation protocols.

Additionally, advanced recipient age was identified as a key risk factor for graft loss, consistent with previous findings in the literature. This highlights the importance of careful recipient selection and the implementation of individualized follow-up strategies to optimize transplant outcomes in this population.

From a clinical perspective, our results suggest that the use of kidneys from AKIN 3 donors could significantly expand the pool of available organs, helping to bridge the gap between organ supply and demand without negatively affecting clinical outcomes. These findings support the need to reconsider current exclusion criteria for these grafts and to develop targeted strategies to optimize their viability and function in routine transplant practice.

Given its retrospective, single-centre nature, modest sample size, and limited statistical power, this study should be viewed as part of an ongoing research effort, providing preliminary insights that require confirmation in larger, multicentre cohorts with extended follow-up.

### 4.7. Limitations

This study presents several limitations that should be considered when interpreting the results. First, no detailed analysis was performed regarding immunological factors and compatibility between donors and recipients. The absence of detailed immunological data, including HLA matching, the presence of donor-specific antibodies (DSA), and the specific immunosuppressive regimens administered to recipients. These variables are critical determinants of rejection risk and long-term graft survival, and their omission limits the interpretation and generalisability of our findings. The absence of a systematic evaluation of HLA matching, the presence of preformed antibodies, and the specific immunosuppressive regimens used in each case prevents a thorough assessment of how these variables may have influenced graft outcomes. Given the critical role of the immune response in graft survival and the occurrence of acute or chronic rejection episodes, this omission represents a significant limitation in the interpretation of our findings.

Another important limitation is the relatively small sample size, which restricts the generalizability of the results. Although a detailed analysis was conducted on recipients of grafts from AKIN 3 donors, the cohort remains modest in size.

Moreover, the retrospective design of the study introduces inherent biases that must be acknowledged. As the analysis was based on previously recorded clinical data, there is a potential for selection bias due to the exclusive inclusion of grafts that met strict selection criteria. Furthermore, the absence of renal injury biomarkers limited our ability to differentiate reversible acute injury from underlying chronic damage and to refine donor selection criteria. The information on procurement methods (e.g., use of machine perfusion or other preservation strategies) was not systematically available, precluding any assessment of their impact on outcomes. The generalisability of our findings is limited by the single-centre Spanish setting and the absence of detailed information on organ procurement practices, including whether mechanical perfusion techniques were used.

Finally, although graft losses appeared to cluster in recipients of kidneys from donors older than 65 years, we were unable to conduct a formal stratified analysis by donor age due to the modest sample size. This observation should therefore be considered hypothesis-generating and explored in larger, adequately powered multicentre studies.

## 5. Conclusions

This single-centre, retrospective, observed that kidney transplantation from carefully selected donors with AKIN stage 3 was associated with comparable medium-term graft function and patient survival to transplants from non-AKI donors, despite a higher incidence of delayed graft function. Given the limited sample size, these results should be interpreted cautiously and regarded as exploratory. Larger, adequately powered multicentre studies, incorporating detailed immunological assessment and long-term follow-up, are warranted to confirm these preliminary observations and to refine donor selection criteria.

## Figures and Tables

**Table 1:** Donor characteristics of study groups.

	AKIN 3 (33)	Non-AKI (33)	*p*
Age at the time of donation (mean)	53.3 ± 12.4	53.1 ± 12.3	0.958
pre-transplant creatinine (mg/dL) (median)	2.01 (IQR: 0.70)	0.85 (IQR: 0.13)	<0.001
eGFR (mL/min/1.73 m^2^) (median)	45.0 (IQR: 16.8)	105.9 (IQR: 12.0)	<0.001
Male % (*n*)	42.4% (14)	51.5% (17)	0.622
Hypertension % (*n*)	21.2% (7)	18.2% (6)	1.000
Diabetes % (*n*)	12.1% (4)	15.2% (5)	1.000
Hypercholesterolemia % (*n*)	18.2% (6)	21.2% (7)	1.000
Smokers % (*n*)	45.5% (15)	33.3% (11)	0.450

Non-AKI: This group includes donors who did not meet diagnostic criteria for acute kidney injury at the time of donation. AKIN3: which defines the AKI group in our study, is characterized by an increase in serum creatinine > 300% from baseline, or an absolute creatinine level ≥ 4.0 mg/dL with an acute rise of at least 0.5 mg/dL, or urine output < 0.3 mL/kg/h for more than 24 h, anuria for more than 12 h, or initiation of renal replacement therapy. *p*-values were calculated using Student’s t-test for normally distributed variables (age) and the Mann–Whitney U test for non-parametric variables (creatinine, eGFR). Current creatinine refers to the last recorded serum creatinine value at the most recent follow-up visit.

**Table 2:** Recipient characteristics of study groups.

	AKIN 3 (57)	Non-AKI (57)	*p*
Age at the time of kidney transplantation. (mean)	56.6 ± 10.7	55.6 ± 10.2	0.594
Current creatinine (mg/dL) (median)	1.39 (IQR: 0.3)	1.28 (IQR: 0.19)	0.144
Male % (*n*)	54.3% (31)	57.9% (33)	0.850
Hypertension % (*n*)	22.8% (13)	26.3% (15)	0.828
Diabetes % (*n*)	19.3% (11)	15.8% (9)	0.806
Hypercholesterolemia % (*n*)	24.6% (14)	17.5% (10)	0.491
Smokers % (*n*)	22.8% (13)	28% (16)	0.667

Non-AKI: This group includes donors who did not meet diagnostic criteria for acute kidney injury at the time of donation. AKIN3: which defines the AKI group in our study, is characterized by an increase in serum creatinine > 300% from baseline, or an absolute creatinine level ≥ 4.0 mg/dL with an acute rise of at least 0.5 mg/dL, or urine output < 0.3 mL/kg/h for more than 24 h, anuria for more than 12 h, or initiation of renal replacement therapy. *p*-values were calculated using Student’s t-test for normally distributed variables (age) and the Mann–Whitney U test for non-parametric variables (creatinine, eGFR). Current creatinine refers to the last recorded serum creatinine value at the most recent follow-up visit.

## Data Availability

The datasets generated and/or analyzed during the current study are not publicly available due to institutional restrictions on patient data confidentiality but are available from the corresponding author on reasonable request. The datasets used and/or analyzed during the current study are available from the corresponding author on reasonable request.

## Abbreviations

AKI	Acute Kidney Injury
AKIN	Acute Kidney Injury Network
DGF	Delayed Graft Function
eGFR	Estimated Glomerular Filtration Rate
IQR	Interquartile Range
CEIm	Comité de Ética de la Investigación con medicamentos
